# Estrogen-Responsive Genes Overlap with Triiodothyronine-Responsive Genes in a Breast Carcinoma Cell Line

**DOI:** 10.1155/2014/969404

**Published:** 2014-01-23

**Authors:** Nancy Bueno Figueiredo, Sílvia Helena Cestari, Sandro José Conde, Renata Azevedo Melo Luvizotto, Maria Teresa De Sibio, Denise Perone, Maria Lúcia Hirata Katayama, Dirce Maria Carraro, Helena Paula Brentani, Maria Mitzi Brentani, Célia Regina Nogueira

**Affiliations:** ^1^Department of Internal Medicine, Botucatu School of Medicine, University of São Paulo State (UNESP), 18618-000 Botucatu, SP, Brazil; ^2^Department of Radiology, Medicine School, University of São Paulo, USP, 01246-903 São Paulo, SP, Brazil; ^3^Research Center, AC Camargo Hospital, 01509-900 São Paulo, SP, Brazil; ^4^Department of Clinical Medicine of University of São Paulo State, Rubiao Junior District, s/n, 18618-000 Botucatu, SP, Brazil

## Abstract

It has been well established that estrogen plays an important role in the progression and treatment of breast cancer. However, the role of triiodothyronine (T_3_) remains controversial. We have previously shown its capacity to stimulate the development of positive estrogen receptor breast carcinoma, induce the expression of genes (PR, TGF-alpha) normally stimulated by estradiol (E_2_), and suppress genes (TGF-beta) normally inhibited by E_2_. Since T_3_ regulates growth hormones, metabolism, and differentiation, it is important to verify its action on other genes normally induced by E_2_. Therefore, we used DNA microarrays to compare gene expression patterns in MCF-7 breast adenocarcinoma cells treated with E_2_ and T_3_. Several genes were modulated by both E_2_ and T_3_ in MCF-7 cells (Student's *t*-test, *P* < 0.05). Specifically, we found eight genes that were differentially expressed after treatment with both E_2_ and T_3_, including *amphiregulin, fibulin 1, claudin 6, pericentriolar material 1, premature ovarian failure 1B, factor for adipocyte differentiation-104, sterile alpha motif domain containing 9, and likely ortholog of rat vacuole membrane protein 1* (fold change > 2.0, pFDR < 0.05). We confirmed our microarray results by real-time PCR. Our findings reveal that certain genes in MCF-7 cells can be regulated by both E_2_ and T_3_.

## 1. Introduction

Most breast cancer risk factors are associated with prolonged exposure of the mammary gland to high levels of estrogen. The biological effects of estrogen are predominantly mediated by two estrogen receptors (ER) that bind to estrogen response elements (EREs) in the promoter region of target genes [1]. Although the involvement of thyroid hormones (TH) in the development and differentiation of normal breast tissue has been established [2–4] and epidemiological and experimental studies have associated TH pathologies with an increased risk of breast cancer, the role of TH remains controversial [5–14]. Vorherr [15] described an increase in the survival of hyperthyroid patients with breast cancer, whereas we have identified a biological link between breast cancer in postmenopausal women and subclinical hyperthyroidism [16]. Most, if not all, major triiodothyronine (T_3_) actions are mediated by specific high affinity nuclear receptors (thyroid receptor, TR), which are encoded by the two genes *THRA* and *THRB* that are also ligand-regulated transcription factors that act via DNA response elements [17]. Recent results have revealed substantial changes in the expression profile of thyroid hormone receptors, suggesting that their deregulation may be involved in breast cancer development [18].

Thyroid receptor is present in both MCF-7 and MDA-MB-231 breast cancer cell lines [19]. We previously demonstrated that T_3_ mimics the effects of estradiol (E_2_) in the ER-positive breast cancer cell line MCF-7, stimulating growth, modulating mRNA transcription of growth factors, and inducing the expression and activity of E_2_-inducible proteins. In addition, these T_3_ effects were antagonized by the simultaneous addition of tamoxifen (TAM), which is a competitive inhibitor of E_2_ that binds to ER. However, we did not observe similar effects in the ER-negative MDA-MB-231 breast cancer cell line, in spite of high amounts of TR. This suggests that in MCF-7 cells both ligands share a common signaling pathway via ER, since the sequence similarity of these hormone receptors allows interactions of TR with EREs or ER with TREs [19]. These results are consistent with those of Zhou-Li et al. (1992), but contradictory to Dinda et al. [20, 21], who found no evidence that T_3_ competitively displaces E_2_ from ER. Recently, Hall et al. [22] showed that both E_2_ and T_3_ promoted proliferation in MCF-7 and T47 cell lines, which was suppressed by coadministration of the ER antagonist fulvestrant (ICI 182780), and T_3_ induced activation of ERE-mediated gene expression (ER*α*, ER*β*, and PR) in MCF-7 cells. They also demonstrated that T_3_ enhanced the effect of E_2_ on cell proliferation in a dose-dependent manner.

We have demonstrated that tamoxifen inhibits transforming growth factor alpha (TGF*α*) gene expression in human breast carcinoma samples treated *in vitro* with T_3_ [23]. These results suggest that T_3_ may play a role in breast cancer development and progression by regulating proliferation, gene expression, and activity of E_2_-inducible proteins such as progesterone receptor (PR) and TGF*α* and indicate an interaction between E_2_ and T_3_ signaling systems.

Here we systematically examined the transcriptional effects of E_2_ and T_3_ in the MCF-7 breast adenocarcinoma cell line using DNA microarrays in order to better understand the actions of these two hormones. We identified their effects on the expression of a large number of genes by using a microarray platform containing 4,608 open reading frame expressed sequence tags (Orestes) [24]. We demonstrated that the expression of eight genes was significantly altered by both E_2_ and T_3_ in MCF-7 cells (fold change > 2.0, positive false discovery rate (pFDR) < 0.05). Out of these eight genes, *amphiregulin* (*AREG*), *fibulin 1 *(*FBLN1*), and *claudin 6* (*CLDN6*) were the most differentially expressed.

## 2. Materials and Methods

### 2.1. Cell Line

MCF-7 cells were grown for 14 days before harvesting in RPMI 1640 supplemented with L-glutamine (2.8 mM), insulin (8 mIU/mL), penicillin-streptomycin (100 U/mL), and 5% charcoal-stripped calf serum (FCS) and kept at 37°C in humidified 5% CO_2_ and air. The medium was changed every two days.

### 2.2. Cell Treatment Conditions

MCF-7 cells were propagated in 150 cm^3^ culture flasks until they reached 40% confluence. Before starting hormone treatments, the medium was replaced with phenol red free RPMI 1640 to eliminate all known sources of estrogen [25]. After 24 h (day 0), the medium was changed and cells were treated in triplicate with 10^−8^ M E_2_ (Sigma-Aldrich, St. Louis, MO, USA, E8874), 10^−8^ M T_3_ (Sigma-Aldrich, T2752), and absolute ethanol (vehicle control) for 72 h. Medium was changed every 24 h. Cells were harvested at the times indicated and cell numbers were counted.

### 2.3. RNA Isolation and Reverse Transcription

Total RNA was extracted from cultured MCF-7 cells by the guanidinium thiocyanate method and analyzed by electrophoresis using 1% agarose gels. One microgram of total RNA was reverse-transcribed with SuperScript III First-Strand Synthesis System for RT-PCR (Invitrogen, Carlsbad, CA, USA, 18080-051).

### 2.4. DNA Microarrays

The microarray glass slide was generated in conjunction with the Laboratório de Biologia Computacional (LBC—Computational Biology Laboratory) at the Hospital do Câncer, São Paulo, Brazil, together with the Laboratório de Análise de Expressão Gênica (LGEA—Gene Expression Analysis Laboratory) at the Instituto Ludwig de Pesquisa sobre o Câncer, São Paulo. The slide contained 4,608 genes from the Human Cancer Genome Project Bank, Instituto Ludwig para a Pesquisa do Câncer, São Paulo. Microarray hybridization and analysis were performed as previously described [26].

### 2.5. Real-Time RT-PCR

Assay-on-Demand Gene Expression Product (Applied Biosystems, Foster City, CA, USA, 4331182), consisting of unlabeled PCR primers and a TaqMan MGB probe (FAM dye-labeled) optimized to work with the TaqMan Universal PCR Master Mix (P/N 4304437) in an ABI Prism 7700 system (Perkin Elmer Life Sciences, Boston, MA, USA), was employed to quantitatively measure *AREG, FBLN1, CLDN6*, and glyceraldehyde-3-phosphate dehydrogenase (*GAPDH*) mRNA expression. All assays were performed in triplicate. mRNA content was normalized to the *GAPDH* mRNA level and differences in expression were determined by the Ct method described in the ABI user's manual (Applied Biosystems).

### 2.6. Statistical Analysis

“Permutation” Student's *t*-test (10,000 permutations) was performed on microarray results without bootstrapping, with a positive false discovery rate (pFDR) less than 0.05, and fold change greater than 2.0.

## 3. Results

**Figure 1:**
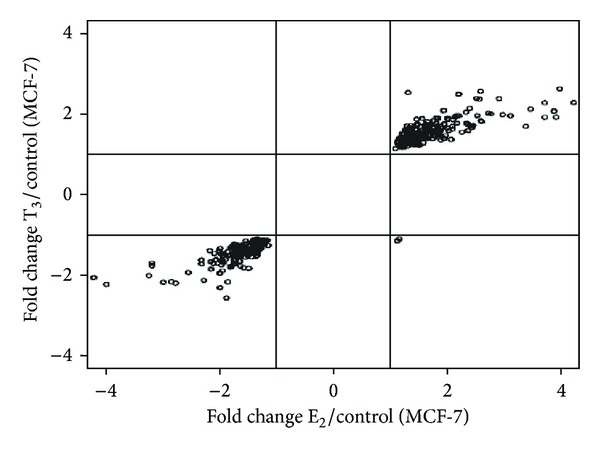
Dispersion of fold changes for the 393 genes modulated by E_2_ and T_3_ in MCF-7 cells. Each point represents the expression of one gene. The dispersion is similar in both treatments for all except two genes.

**Figure 2:**
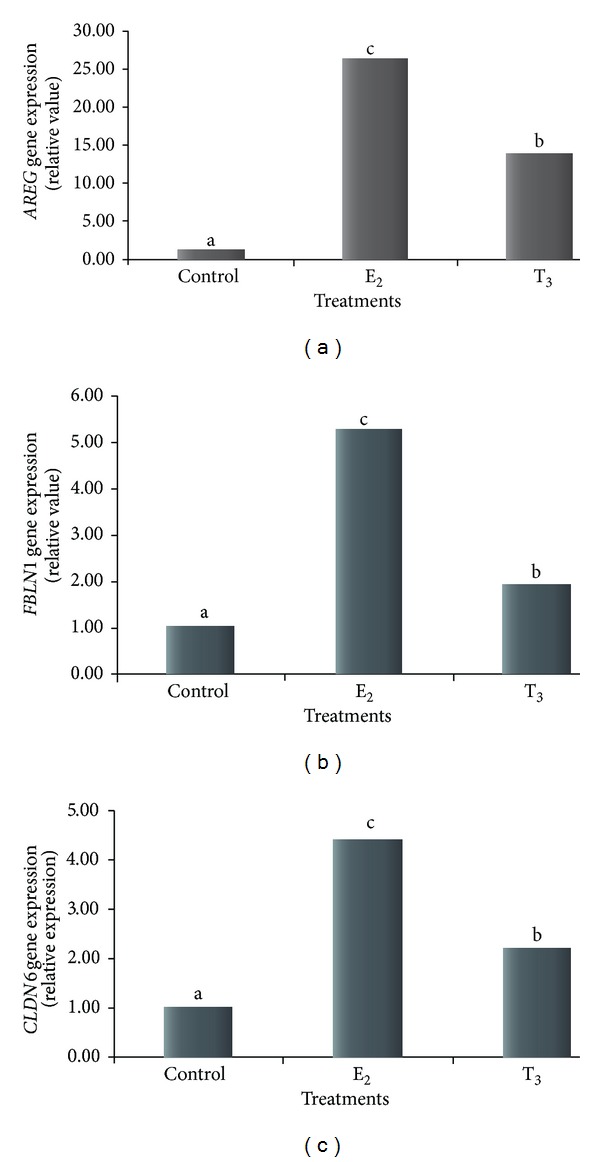
(a) Expression of *AREG* gene analyzed by real-time PCR in MCF-7 cells. (b) Expression of *FBLN1* gene analyzed by real-time PCR in MCF-7 cells. (c) Expression of *CLDN6* gene analyzed by real-time PCR in MCF-7 cells. Results are expressed relative to the expression in the control cells for E_2_-treated (10^−8 ^M) and T_3_-treated (10^−8 ^M) MCF-7 breast carcinoma cells. The use of different letters indicates that there was a statistical difference of *P* < 0.05.

The influence of T_3_ on the gene expression profile of MCF-7 cells was examined and compared to the effects of treatment with E_2_. RNA samples extracted from triplicate samples of MCF-7 cells after 24 h of E_2_ or T_3_ treatment were analyzed for E_2_- or T_3_-regulated gene expression by comparing to cells treated with vehicle control. Genes with *P* < 0.05 (Student's *t*-test) in paired group comparisons were considered as differentially expressed. We verified that 393 genes were differentially expressed after both treatments (up- or downregulated) (Figure 1). After applying a 2-fold change as a cut-off point and a pFDR < 0.05, MCF-7 cells treated with E_2_ displayed 39 genes that were differentially expressed compared to the control, whereas after T_3_ treatment only 25 genes were differentially expressed relative to control cells. Eight genes were commonly modulated after treatment with both E_2_ and T_3_ (Table 1), although the response to T_3_ was less pronounced than to E_2_. Out of these eight genes, *AREG, FBLN1*, and *CLDN6 *were strongly expressed after treatment with both T_3_ and E_2_. In order to confirm our microarray data, we quantified *AREG, FBLN1*, and *CLDN6* mRNAs using quantitative RT-PCR. Treatment with both E_2_ and T_3_ at 10^−8 ^M resulted in overexpression of *AREG* (26.3- and 13.8-fold more, resp.), *FBLN1* (5.3- and 1.9-fold more, resp.), and *CLDN6* (4.4- and 2.2-fold more, resp.) (Figure 2).

## 4. Discussion

In breast cancer, several clinicopathological markers are frequently used alone or in combination to assess patient risk. For example, lymph-node stage, tumor size, and histologic grade are important elements of the major prognostic indices, whereas ER status is widely regarded as the primary predictor of response to hormonal (antiestrogen) therapy. Microarray data sets from large studies of breast carcinomas have revealed several underlying signatures associated with the primary physiology of the tumor that have important prognostic and predictive implications.

In previous studies, we have demonstrated that T_3_, in supraphysiological doses, is able to increase the progression of ER-positive breast cancer, enhancing the expression of genes normally stimulated by E_2_ and suppressing genes normally inhibited by E_2_ [19]. Based on those results, we sought to identify genes that are influenced by both hormones in order to identify additional markers of progression.

Many genes were equally modulated by E_2_ and T_3_ in MCF-7 breast carcinoma cells (Student's *t*-test, *P* < 0.05) (Figure 1). Using more stringent criteria (2-fold cutoff and pFDR < 0.05), the number of genes modified by E_2_ and T_3_ decreased to eight. Out of these eight genes, *AREG, FBLN1*, and *CLDN6* were strongly expressed by both T_3_ and E_2_ treatment. We validated our microarray results using real-time PCR on *AREG*, *FBLN1*, and *CLDN6* expression (Figure 2).

*Amphiregulin *(*AREG*), the most differentially expressed gene, codes for a glycoprotein, that is, a member of the epidermal growth factor family (EGF), the members of which are ligands that can bind EGFR. *AREG* was discovered in concentrated conditioned medium from MCF-7 cells [27]. Numerous studies have sought to characterize the transcriptional network associated with estrogen response using cell culture experiments. Kenney et al. [28] inserted pads with recombinant *AREG* in the breast of oophorectomized rats, which reestablished the premature development of the ductal mammary epithelium and prompted hyperplasia. Therefore, *AREG* seems to play an intermediary role in maturing the mammary gland and in stimulating the initiation of mammary oncogenesis. Martinez et al. [29] treated MCF-7 cells with 10^−9 ^M E_2_, 10^−9 ^M E_2_ with 10^−6 ^M TAM (E_2_ + TAM), TAM alone, and vehicle control for 24 hours. They observed that adding TAM to the treatment with E_2_ decreased *AREG* mRNA expression by 38%, suggesting that E_2_ stimulates *AREG* expression via ER. Using microarrays, Frasor et al. [30] observed an upregulation of *AREG* in MCF-7 cells treated with E_2_, which was reversed by tamoxifen. When Vendrell et al. [31] treated MVLN cells, a breast carcinoma cell line derived from MCF-7 cells, for 4 days with E_2_, they observed that *AREG* was one of the differentially expressed genes using cDNA miniarrays. A recent microarray analysis identified E_2_-regulated genes in a model in which human breast tissue was transplanted into mice, which were then treated with estradiol [32]. Interestingly, *AREG* was the most upregulated gene. Several studies have examined *AREG* expression in breast carcinomas by immunohistochemistry and found that *AREG* expression is higher in infiltrative breast carcinoma than in normal epithelium and is associated with regional lymph-node metastasis [33–35]. In addition, *AREG* upregulates the expression of genes associated with invasion [27]. Modulation of the *AREG* gene by T_3_ has not been previously reported. While the increase in *AREG* expression in MCF-7 cells after T_3_ treatment that we found was lower than the increase after E_2_ treatment, it was highly significant. Thus, *AREG* appears to be an important target gene for E_2_ and T_3_ in MCF-7 breast cancer cells.

*Fibulin *1 (*FBLN1*) is the prototype member of the fibulin family of ECM proteins and binds to many ECM proteins, including fibronectin, laminin-1, fibrinogen, aggrecan, and versican. Fibulin 1 may be involved in cell motility and anchorage-independent growth of tumor cells [36] and is overexpressed in breast cancer specimens and breast cancer cell lines [37–39]. *FBLN1 *was previously reported to be induced by E_2_ in MCF-7 cells [40] and in ER-positive ovarian cancer cell lines [41]. We have shown for the first time that *FBLN1* is upregulated in breast cancer cell lines treated with T_3_.

*Claudin 6 *(*CLDN6*), a member of the Claudin family, is involved in the formation of the GAP junction [42]. While Offner et al. [43] reported the expression of *CLDN6* in breast cancer cell lines, its role in carcinogenesis remains controversial [44–46]. Wu et al. [47] noted that cells with a high level of expression of claudin 6 grew slowly and had a higher rate of death than control cells, suggesting that claudin 6 may function as a cancer suppressor. Its downregulation may contribute to the malignant progression of certain types of breast cancers [48]. To our knowledge, this is the first study showing that E_2_ and T_3_ modulate *CLDN6* expression in a breast cancer cell line.

Some of the other genes that we identified as being regulated by E_2_ and T_3_ are directly involved in cell proliferation, such as *EMP1*,* IFNAR2*,* VMP, FLJ20073*,* MYC*, and *AREG*, and some are involved in nucleotide binding and/or protein binding, such as *PFKFB3*,* APPBP2*,* SSFA2*, and *NALP7*. Other genes, such as *CSTA*, show altered expression in the tissue invasion process during breast carcinogenesis [49]. Expression of *BRAP*, responsible for ubiquitination of the product of the tumor suppressor gene *BRCA1*, was increased in our study and in other studies [50] and *FBLN1* and *ADAM9* are associated with migration and tissue invasion [51, 52]. We also identified genes that had not been previously correlated with breast cancer, including *TERF2IP*, which is involved in telomere regulation [53], *IGSF1*, which is involved in intracellular adhesion and transcription and is a signal transduction regulator [54], *NMT2*, which acts in the protein myristoylation process [55], FAD104, which positively regulates fat cell differentiation [56], *PCM1*, involved in centromere amplification and genomic instability [57], *VWF*, involved in cellular adhesion [58], *C1R*, which is involved in immune response and complement system activation [59], and *POF1B*, which binds nonmuscle actin filaments [60].

This is the first report of T_3_ modulation of *FBLN1, CLDN6*, and *AREG*. The magnitude of the increase in expression of *AREG*, *FBLN1*, and *CLDN6* in MCF-7 cells treated with T_3_ was less than when treated with E_2_ but highly significant. This may be because these genes have more binding consensus sequences for E_2_ than T_3_ receptors. Specifically, within 5,000 bp upstream of the transcriptional initiation site, *AREG* has 10 EREs and 8 TREs, *FBLN1* has 6 TREs and 6 EREs, and *CLDN1* has 12 EREs and 10 TREs.

## 5. Conclusion

We have shown that T_3_ treatment results in a gene expression pattern similar to E_2_ treatment, up- or downregulating a group of the same genes, suggesting that these two hormones can cause similar phenotypes. Our *in vitro* observation suggests a molecular mechanism by which thyroid hormone can be a relevant factor for breast cancer progression through the induction of genes involved in growth and invasion.

## Figures and Tables

**Table 1:** Genes significantly modulated by E_2_ and T_3_ in MCF-7 cells.

Symbol	Gene	Biological process	Fold change (adj. *P* value)	
			E_2_	T_3_
*AREG *	Amphiregulin, colorectum cell-derived, growth factor	Epidermal growth factor	18.13	3.63
*FAD104 *	Fibronectin type III domain containing 3B	Positive regulation of fat cell differentiation	3.89	2.04
*FBLN1 *	Fibulin 1	Interspecies interaction between organisms	2.60	2.53
*POF1B *	Premature ovarian failure, 1B	Protein encoded by this gene binds nonmuscle actin filaments	2.58	2.34
*CLDN6 *	Claudin 6	Calcium-independent cell-cell adhesion	2.53	2.36
*PCM1 *	Pericentriolar material 1	G2/M transition of mitotic cell cycle/microtubule anchoring	2.38	2,02
*FLJ20073 *	Sterile alpha motif domain containing 9	Regulation of cell proliferation and apoptosis	2.22	2.46
*VMP1 *	Likely ortholog of rat vacuole membrane protein 1	Cell-cell adhesion	2.41	2.11

Fold: expression difference; *P* value: significance value of Student's *t*-test; FDR: false discovery rate.
